# Differential expression of aerobic oxidative metabolism-related proteins in diabetic urinary exosomes

**DOI:** 10.3389/fendo.2022.992827

**Published:** 2022-09-14

**Authors:** Tianci Liu, Yizhao Wang, Man Zhao, Jun Jiang, Tao Li, Man Zhang

**Affiliations:** ^1^ Clinical Laboratory Medicine, Beijing Shijitan Hospital, Capital Medical University, Beijing, China; ^2^ Beijing Key Laboratory of Urinary Cellular Molecular Diagnostics, Beijing, China; ^3^ Clinical Laboratory Medicine, Peking University Ninth School of Clinical Medicine, Beijing, China

**Keywords:** diabetes, urine, exosomes, aerobic oxidation, markers

## Abstract

**Background:**

As a metabolic disease, any abnormality in the aerobic oxidation pathway of glucose may lead to the occurrence of diabetes. This study aimed to investigate the changes in proteins related to aerobic oxidative metabolism in urinary exosomes of diabetic patients and normal controls of different ages, and to further verify their correlation with the pathogenesis of diabetes.

**Methods:**

Samples were collected, and proteomic information of urinary exosomes was collected by LC-MS/MS. ELISA was used to further detect the expression of aerobic and oxidative metabolism-related proteins in urinary exosomes of diabetic patients and normal controls of different ages, and to draw receiver operating characteristic (ROC) curve to evaluate its value in diabetes monitoring.

**Results:**

A total of 17 proteins involved in aerobic oxidative metabolism of glucose were identified in urinary exosome proteins. Compared with normal control, the expressions of PFKM, GAPDH, ACO2 and MDH2 in diabetic patients were decreased, and the expression of IDH3G was increased. The concentrations of PFKM, GAPDH and ACO2 in urinary exosomes were linearly correlated with the expression of MDH2 (P<0.05). These four proteins vary with age, with the maximum concentration in the 45-59 age group. PFKM, GAPDH, ACO2, and MDH2 in urinary exosomes have certain monitoring value. When used in combination, the AUC was 0.840 (95% CI 0.764-0.915).

**Conclusions:**

In diabetic patients, aerobic oxidative metabolism is reduced, and the expression of aerobic oxidative metabolism-related proteins PFKM, GAPDH, ACO2, and MDH2 in urinary exosomes is reduced, which may become potential biomarkers for monitoring changes in diabetes.

## Introduction

Diabetes mellitus (DM) is a common metabolic disorder with hyperglycemia as the main symptom (1). According to statistics, the number of adults with diabetes worldwide has reached 537 million in 2021, and by 2045, the number of people with diabetes is expected to reach 783 million (2), a serious threat to human life and health. Diabetes is caused by a variety of factors, and it is currently believed that insulin resistance and impaired β-cell secretory function are the most important factors (3, 4). Most pharmacological interventions for the treatment of diabetes rely on promoting insulin secretion or supplementing insulin to regulate blood sugar.

Extracellular vesicles (EVs) include microvesicles, apoptotic bodies, and exosomes (5). Among them, exosomes have a lipid bilayer membrane with a diameter of 30-150 nm, which can be secreted by most cells of the body. Exosomes were first discovered in 1983. Johnstone et al. found that the formation of some extracellular vesicles was the mechanism for the loss of transferrin receptors in mature red blood cells, and these vesicles were named exosomes (6). The formation of exosomes includes the double invagination of the plasma membrane and the formation of intracellular multivesicular bodies of intraluminal vesicles (7). Exosomes have a double-membrane structure and their contents include various types of proteins, DNA, and RNA (mRNA, miRNAs, and other small regulatory RNAs, etc.) (8, 9). Exosomes can be detected in most body fluids, including blood, tears, urine, and milk.

Exosomes are secreted by different cell types under normal and pathological conditions, and their contents and functions vary accordingly (10). The massive contents carried by exosomes can mediate intercellular communication and signal transduction (11), and play an indispensable role in information transfer between cells and organs. Exosomes are widely involved in physiological and pathological processes such as growth and development (12, 13), energy metabolism (14), and immune regulation (15), and provide potential markers for the diagnosis of a series of diseases. It has been found that exosomes affect insulin secretion and the regulation of insulin resistance through various pathways. This is of great significance for the early diagnosis, treatment, and prognosis evaluation of diabetes (16–18).

Normal blood glucose levels are maintained at a relatively constant level. The aerobic oxidation pathway of glucose is the basis and connecting hub for communicating the metabolic pathways of glucose, lipids and proteins. The large amount of ATP produced by aerobic oxidation provides daily energy. This is very important to ensure the normality of various tissues and organs of the human body, especially the brain tissue, which is almost completely dependent on glucose for energy for neural activities. The maintenance of blood glucose concentration is closely related to glucose metabolism. The aerobic oxidative metabolism of glucose is closely related to the occurrence and development of diabetes and its complications (19). The key to revealing the pathogenesis of diabetes is to clarify the expression changes and influence of a series of catalytic enzymes of glucose aerobic oxidative metabolism in diabetic patients.

Glucose metabolism in normal people changes with age because insulin secretion and insulin sensitivity are impaired with age (20, 21). However, the expression changes of aerobic oxidative metabolism-related proteins are still not particularly clear. Especially as urine is a collection of systemic metabolites, how the expression changes of aerobic oxidative metabolism-related proteins changes in the urinary exosomes of diabetic patients and normal people of different ages has attracted our attention.

In this study, based on exosomes, a statistical analysis was performed on the quantitative values of proteins related to glucose aerobic oxidative metabolism in urinary exosome proteomics. Then, the expression changes of aerobic oxidation-related proteins in diabetic patients and normal controls of different ages were explored to further verify their correlation with the incidence of diabetes. And evaluate its diagnostic and monitoring efficacy for diabetes, so as to provide suitable, sensitive and specific biomarkers for diabetes monitoring.

## Materials and methods

### Patients

A total of 20 patients with diabetes mellitus (DM) were selected from Peking University Ninth School of Clinical Medicine between October 2020 and March 2021. In Total, 20 healthy people matched for age and sex were selected as the control group, and clinical data were retrospectively collected. In diabetic patients, fasting blood glucose (FBG) ≥7.0 mmol/l, glycosylated hemoglobin (HbA1c) ≥6.5%, or OGTT 2-hour blood glucose ≥11.1 mmol/L. People with normal physical examination results were selected as the normal control group, excluding hypertension and diabetes mellitus. All subjects were free of hematuria, proteinuria, and ketosis, and diseases such as urinary system diseases and tumors were excluded. This experiment was approved by the Ethics Committee of Beijing Shijitan Hospital. All subjects have provided informed consent before inclusion in this study and collection of specimens, and all procedures were performed in accordance with the ethical standards of the Declaration of Helsinki.

### Exosome extraction

30 mL of clean morning urine from subjects was collected, and the obtained urine samples were centrifuged at 4°C and processed at 1500 g × 10 min first and then at 10000 g × 30 min to remove cells and debris. Then, the supernatant was filtered through a 0.22 μm filter (Millipore, SLGVR33RB). Urine was concentrated by ultrafiltration tubes (Millipore, UFC910024) and exosomes were collected using size exclusion SEC (qEV10/35nm, IZON, Shanghai, China) (22–24). Finally, the urinary exosomes were resuspended in approximately 1 ml PBS and stored directly at -80°C until use.

### Identification of urinary exosomes

According to the guidelines of the International Society of Extracellular Vesicles (ISEV) for the characterization of exosomes, the morphology of exosomes was detected by transmission electron microscope (TEM), the size and concentration of exosomes were identified by nanoparticle tracking analysis (NTA), and exosome markers were analyzed by western blot analysis according to the expression of exosome markers.

**TEM**: The urine exosome sample was dropped on the sample-carrying copper grid, and after standing at room temperature for 5 minutes, the liquid was blotted dry from the side of the filter screen with filter paper. Then, a drop of 2% uranyl acetate was dropped on the sample, incubated for 1 min at room temperature, and the surface liquid was blotted with filter paper. After drying at room temperature, the morphology of urinary exosomes was observed under a microscope.

**NTA**: The samples were diluted to the appropriate concentration with precooled 1×PBS and injected into the Nanoparticle Tracking Analyzer NTA (ZetaVIEW S/N 17-310, PARTICLE METRIX) with a 1 mL syringe for detection. Urinary exosome concentration results for NTA are uploaded as **Supplementary Materials 1**.

**Western Blot**: To further validate the results of the urinary exosome proteome analysis, we performed western blot experiments. After exosome lysis, the same amount of protein was loaded onto a 12% Tris-HCl SDS-polyacrylamide gel, and transferred to PVDF membrane by Trans-Blot Turbo, and then blocking solution was added and shaken at room temperature for two hours. Primary antibody CD9 (Abcam, ab236630) and primary antibody CD63 (Abcam, ab271286) were diluted 1:1000 and incubated overnight in the refrigerator. After washing 3 times in TBST for 15 min, horseradish peroxidase-conjugated secondary antibody (1:3000 dilution; Bioss, Beijing, China) was added, incubated at room temperature for 2 h, washed 3 times in TBST, and then chemiluminescent (ECL) exposure was performed.

### Urine exosome mass spectrometry

We prepared mobile phase solution A (100% MS water, 0.1% formic acid) and solution B (100% acetonitrile, 0.1% formic acid). Peptides were separated in an analytical column using a linear gradient elution method. The mass spectra were analyzed by a QExactive HF-X mass spectrometer (Thermo Fisher) using data independent acquisition (DIA) mode with a full scan range of m/z 350-1500 and a resolution of 120,000 (m/z 200). The automatic gain control (AGC) target value was 2 × 10^5^, the NanosprayFlex™ (ESI) ion source, and the ion spray voltage was set to 2.4 kV. MS/MS spectral results were queried in the SwissProt human database within Uniprot (www.UniProt.org) using the proteome discovery software suite (Thermo Fisher Scientific v2.1). At the protein level, a 1% false discovery rate (FDR) was used as a filter and each protein contained at least one unique peptide. A fold change>1.5 and P value<0.05 were considered significant differences based on unique peptide results and proteins with fold changes. The STRING database was then used to search for associations between interacting genes/proteins and predicted proteins.

### ELISA

Urinary exosomes from diabetic patients and normal controls were used as validation specimens. Urine exosome samples were lysed with RIPA strong lysis buffer, and the protein concentration was measured. The total amount of immobilized protein was 10μg, and the sample volume was adjusted to 100μl with sample buffer. The protein concentrations related to aerobic oxidative metabolism of glucose in urinary exosomes were determined using an ELISA kit from Wuhan Fine Biotechnology (Wuhan, China). According to the instructions, 100μl of the sample to be tested was added to each well, and incubated at 37°C for 90 minutes, and after washing the plate, the plate was incubated with biotin-labelled antibody working solution at 37°C for 60 minutes. The plate was then washed thoroughly and then the HRP-streptavidin conjugate was added and incubated for 30 minutes. Then, the plate was washed, TMB substrate solution was added, the stop solution was added after 20 minutes, and the absorbance was measured at 450nm with a microplate reader. Each sample was measured 3 times, and the unknown sample concentration was calculated according to the standard curve. The target protein expression in the obtained exosomes was defined as ng/10 μg, representing the target protein content per 10 μg of total protein.

### Statistics

All experimental data are expressed as the mean ± standard deviation (SD) or median (lower quartile-upper quartile). Statistical analysis was performed using GraphPad Prime 8.0 (GraphPad, La Jolla, CA, USA) software, and differences between groups were compared using two independent samples t-test. Correlation analysis was determined using Person correlation. P < 0.05 was considered significant, with statistical significance. Associations between interacting genes/proteins and predicted proteins were searched using the STRING database v11.0.

## Results

### Clinical characteristics

The discovery cohort trial included urine exosome samples from 20 diabetic (DM) patients and 20 normal controls (NC), and the validation cohort included 52 diabetic (DM) patients and 55 normal controls (NC). Age and Gender-matched, the clinical data and related indicators of the selected subjects were shown in **Table 1**. And there was no statistical difference between the diabetes group and the normal control group in terms of serum total cholesterol (TC), aspartate aminotransferase (AST), alanine aminotransferase (ALT), etc. (P>0.05).

**Table 1:** Demographic and clinical characteristics of diabetic patients (DM) and normal control (NC).

Characteristics	Discovery cohort			Validation cohort		
	DM (n = 20)	NC (n = 20)	*P* value	DM (n = 52)	NC (n = 55)	*P* value
Age, years	53.45 ± 0.82	51.90 ± 0.83	ns	55.67 ± 1.14	52.98 ± 0.89	ns
Gender; Male/female	10/10	10/10	NA	26/26	28/27	NA
BMI	25.08 ± 0.51	23.22 ± 0.44	**	25.75 ± 0.43	22.84 ± 0.22	***
WBC×10^9^/L	6.33 ± 0.40	5.53 ± 0.40	ns	6.23 ± 0.22	5.65 ± 0.19	ns
RBC×10^12^/L	4.80 ± 0.10	4.79 ± 0.08	ns	4.98 ± 0.07	4.82 ± 0.05	ns
Hb, g/L	146.50 ± 3.92	142.00 ± 4.24	ns	150.70 ± 2.29	145.00 ± 1.94	ns
NE, %	55.53 ± 1.55	58.23 ± 2.18	ns	56.81 ± 0.88	57.73 ± 0.94	ns
LY, %	35.51 ± 1.59	32.80 ± 1.85	ns	34.33 ± 0.86	33.39 ± 0.87	ns
TC, mmol/L	5.11 ± 0.26	4.56 ± 0.11	ns	4.93 ± 0.14	4.62 ± 0.10	ns
AST, U/L	22.50 ± 1.53	19.36 ± 0.63	ns	26.94 ± 3.15	20.80 ± 0.61	ns
ALT, U/L	24.30 ± 1.57	20.90 ± 1.46	ns	19.44 ± 1.70	22.16 ± 1.27	ns
ALB, g/L	43.93 ± 0.70	42.77 ± 0.83	ns	44.36 ± 0.37	44.23 ± 0.39	ns
FBG, mmol/L	8.11 ± 0.68	5.23 ± 0.44	**	11.28 ± 1.50	5.88 ± 0.15	***
HbA1c, %	7.29 ± 0.40	5.98 ± 0.31	**	9.40 ± 0.15	5.43 ± 0.06	***
CEA, ng/mL	2.23 ± 0.19	2.21 ± 0.14	ns	2.13 ± 0.11	2.54 ± 0.19	ns
AFP, ng/mL	3.37 ± 0.40	3.29 ± 0.23	ns	3.35 ± 0.22	3.49 ± 0.20	ns

BMI, Body Mass Index; WBC, White blood cell; RBC, Red blood cell; Hb, Hemoglobin; NE, Neutrophils; LY, Lymphocytes; TC, Serum total cholesterol; AST, Aspartate aminotransferase; ALT, Alanine aminotransferase; ALB, Albumin; FBG, Fasting Blood Glucose; CEA, Carcinoembryonic antigen; AFP, Alpha fetoprotein. P value: ns, no significance; NA, Not Available; **, P<0.01; ***, P<0.001.

### Characteristics of exosomes derived from urine

**Figure 1:**
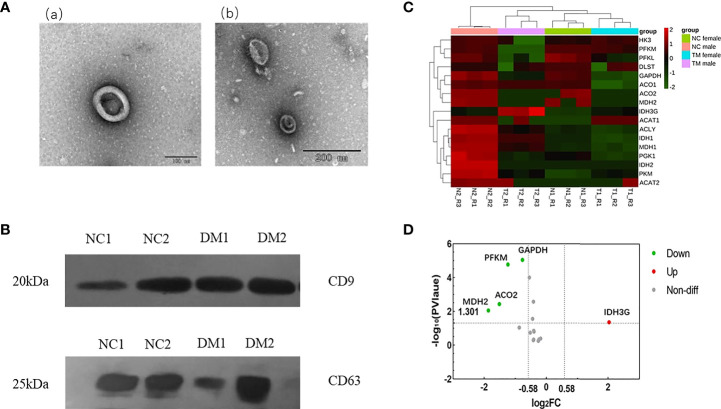
Identification of urinary exosomes and proteomic analysis of enzymes related to aerobic oxidative metabolism. **(A)** Representative TEM image of urinary exosomes. Exosomes with oval morphology are shown (scale bars; a = 100 nm, b = 200 nm). **(B)** Western blot images of urinary exosome markers CD9, CD63. Significant expression of CD9 and CD63 markers could be seen in NC group and DM group. **(C)** Hierarchical clustering heat map analysis of aerobic oxidative metabolism related proteins in diabetic group (DM) and normal control group (NC). N1, female in NC group; N2, male in NC group; T1, female in DM group; T2, male in DM group. **(D)** Volcano analysis of aerobic oxidative metabolism related proteins in diabetic group (DM) and normal control group (NC).FC, fold change; The abscissa is represented by log2 (FC), the genes with greater differences are distributed at both ends, and the ordinate is represented by -log10 (P value).

We isolated exosomes from the urine of diabetic patients and normal people, and used TEM to detect the morphology of exosomes. As shown in **Figure 1A**, we can see the obvious double-membrane oval shape, and the size range of exosomes was 110~130nm. In addition, we measured by NTA, the average size of exosomes was 126.3 nm (uploaded as **Supplementary Materials 1**). To further confirm that the exosomes had been isolated, we used western blot to detect the presence of two exosome markers: the transmembrane proteins CD9 and CD63 (**Figure 1B**). As shown, exosomes isolated from the urine of both DM and NC groups expressed CD9 and CD63. Collectively, these results demonstrate the existence of exosomes.

### Expression analysis of aerobic oxidative metabolic proteins in urinary exosomes

Urinary exosome protemic data were analyzed, and the false discovery rate (FDR) was set to 1%. A total of 17 aerobic oxidative metabolism-related proteins were identified in urinary exosome proteins (**Table S1**), and the hierarchical clustering heat map analysis is shown in **Figure 1C**. The expression of these proteins in urinary exosomes varied. It is considered that fold change>1.5, P value<0.05 has a significant difference, and the volcano plot is shown in **Figure 1D**. Compared with normal controls, the expression of 4 proteins was decreased, including ATP-dependent 6-phosphofructokinase (muscle type; PFKM), Glyceraldehyde-3-phosphate dehydrogenase (GAPDH), Aconitate hydratase (mitochondrial; ACO2), Malate dehydrogenase (mitochondrial; MDH2). In addition, the expression of Isocitrate dehydrogenase [NAD] subunit gamma (mitochondrial; IDH3G) was increased.

### Functional analysis of aerobic oxidative metabolic proteins in urinary exosomes

**Figure 2:**
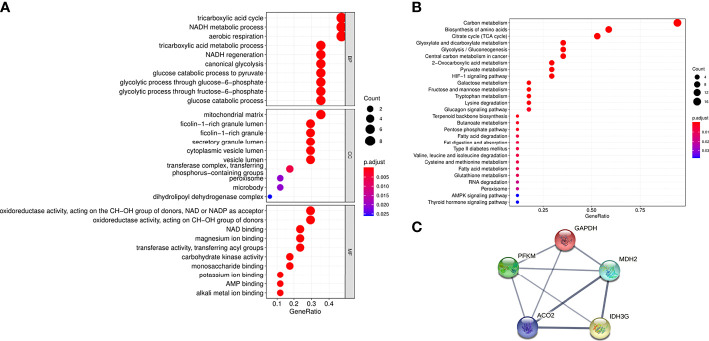
Functional analysis of aerobic oxidative metabolism related proteins in urinary exosomes. **(A)** GO enrichment analysis of 17 aerobic oxidative metabolism related proteins. The ordinates represent GO functional categories: biological process (BP), molecular function (MF), and cellular component (CC). The horizontal axis represents the proportion of protein, the size of the dot represents the number of genes, and the color represents the size of the p value. **(B)** KEGG enrichment analysis of 17 aerobic oxidative metabolism related proteins. The vertical axis represents the significantly enriched KEGG pathways, the horizontal axis represents the proportion of proteins, the size of the dots represents the number of genes, and the color represents the size of the p value. **(C)** PPI network of 5 differential proteins in diabetic patients and normal controls. The thickness of the line indicates the strength of the correlation.

To further understand the functions of the 17 proteins, GO and KEGG analyses were performed in this study. Biological function analysis showed that these proteins were closely related to the tricarboxylic acid cycle, NADH metabolic process, and aerobic oxidation. Most of the cellular components were located on the mitochondrial matrix, and their molecular functions were mostly related to oxidoreductase activity (NAD and NADH synthesis) (**Figure 2A**). Through KEGG pathway analysis, carbon metabolism, amino acid biosynthesis, and the tricarboxylic acid cycle were the most abundant functions (**Figure 2B**). At the same time, this study used the STRING database to predict the interaction analysis of five differential proteins, as shown in **Figure 2C**, these proteins have strong interactions.

### Changes in the expression of aerobic oxidative metabolic proteins in urinary exosomes in different age groups

**Figure 3:**
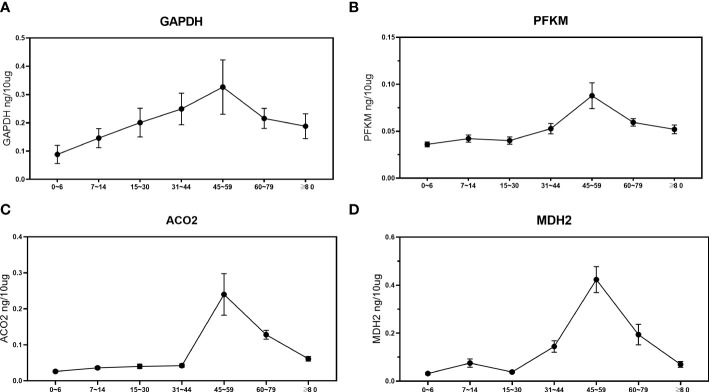
Analysis of the expression changes of four proteins in urinary exosomes of healthy people of different ages. Divided into 7 groups, 20 in each group, a total of 140 samples. Figure shows the concentration changes of GAPDH **(A)**, PFKM **(B)**, ACO2 **(C)** and MDH2 **(D)** in order. The abscissa represents different age groups, and the ordinate represents the expression of each protein measured by ELISA. The unit is ng/10μg, which represents the target protein content per 10μg total protein.

Taking healthy people of different ages as the research objects, the expression trends of four aerobic oxidative metabolism-related proteins with reduced expression in urinary exosomes were analyzed by ELISA. Combined with the age classification standards of the United Nations World Health Organization, seven groups were divided, with 20 people in each group, including 0~6 years old, 7~14 years old, 15~30 years old, 31~44 years old, 45~59 years old, 60~ 79 years old and ≥80 years old. Clinical information of healthy people of different age groups is uploaded as **Supplementary Materials 2**. As shown in **Figure 3**, the expression levels of PFKM, GAPDH, ACO2, and MDH2 in the urinary exosomes of healthy people during childhood were relatively low, and gradually increased with age. The expression level was highest at 45-59 years old, and then gradually decreased, showing a peak trend.

### Correlation analysis of catalytic enzymes for aerobic oxidative metabolism in urine exosomes

**Figure 4:**
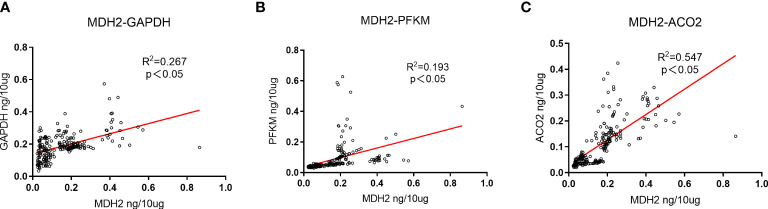
Expression correlation analysis of GAPDH, PFKM, ACO2 and MDH2. **(A)** The correlation between the expression changes of GAPDH in urinary exosomes and MDH2. R2 = 0.267, P<0.05, there is a significant positive correlation. **(B)** The correlation between the expression changes of PFKM in urinary exosomes and MDH2. R2 = 0.193, P<0.05, showing a positive correlation. **(C)** The correlation between the expression changes of ACO2 in urinary exosomes and MDH2. R2 = 0.547, P<0.05, showing a strong positive correlation. The unit is ng/10μg, which represents the target protein content per 10μg total protein.

PFKM, GAPDH, ACO2, and MDH2 are important catalytic enzymes related to aerobic oxidative metabolism, and there is a certain correlation among them. MDH2 is a metabolic enzyme in the steps of the tricarboxylic acid cycle. Based on the ELISA results, we further explored the correlation of PFKM, GAPDH, ACO2 and MDH2 expression in urinary exosomes, as shown in **Figure 4**. The concentrations of aerobic oxidative metabolism catalytic enzymes PFKM, GAPDH and ACO2 in urinary exosomes were significantly linearly related to the expression of MDH2 (P<0.05).

### Differential expression of aerobic oxidative metabolic proteins in urinary exosomes of diabetic patients

**Figure 5:**
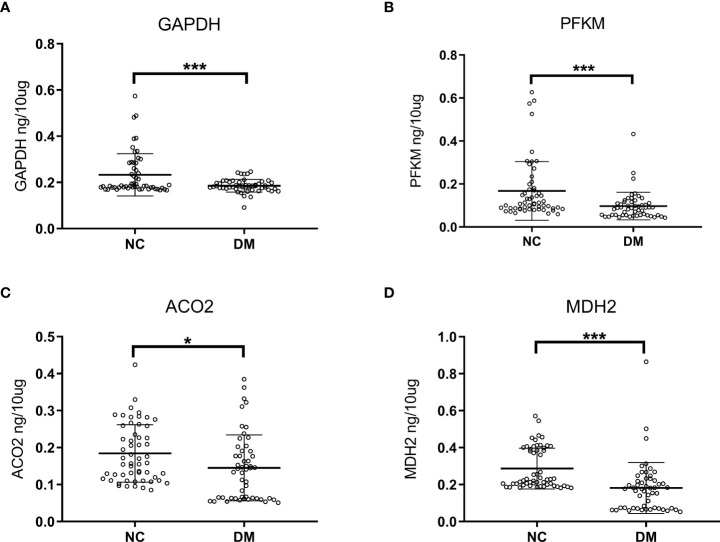
Analysis of ELISA results in diabetic group (DM, n=52) and normal controls (NC, n=55). Concentrated expression of GAPDH **(A)**, PFKM **(B)**, ACO2 **(C)** and MDH2 **(D)** in urinary exosomes was measured by ELISA. The expressions of GAPDH, PFKM, ACO2 and MDH2 in the urine of the diabetic patients were lower than those of the normal control group. The unit is ng/10mg, which represents the target protein content per 10mg total protein. Symbols represent individual subjects, each measured once in an independent experiment, ***, p<0.001; *, p<0.05.

Considering the actual situation, we performed ELISA experiments on the four proteins whose expression was reduced, including PFKM, GAPDH, ACO2, and MDH2. The protein concentration was calculated by the standard curve, and the validation cohort included the diabetic group (52 cases) and the normal control group (55 cases), as shown in **Figure 5**. The expression levels of four proteins in urinary exosomes of diabetic group (DM) were significantly lower than those of normal control group (NC), and the difference was statistically significant (P<0.05). At the same time, we analyzed the differential proteins between males and females in the diabetic group (52 cases) and normal control group (55 cases). The relevant results were uploaded as **Supplementary Materials 3**. As shown in the figure, no significant difference was found in the four proteins between diabetic men and diabetic women (*p*>0.05). The results of the normal control group were the same, and no significant difference in expression between males and females was found.

### Oxidative metabolism-related proteins in urinary exosomes have clinical value for auxiliary monitoring of diabetes mellitus

**Figure 6:**
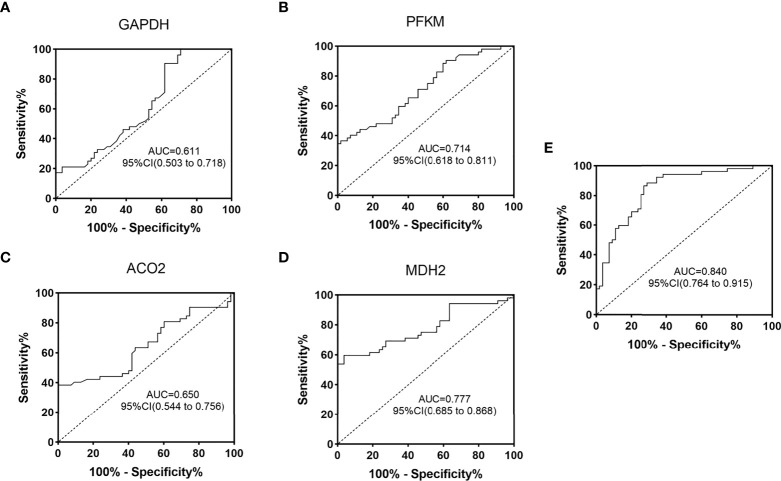
ROC curve analysis of oxygenation-related proteins in urinary exosomes in monitoring of diabetic patients. ROC curves were drawn according to the protein concentration in the urine exosomes of diabetic group (DM) and normal control group (NC) measured by ELISA. GAPDH **(A)**, PFKM **(B)**, ACO2 **(C)**, MDH2 **(D)** and the combined ROC curve of the four proteins **(E)**. AUC, area under the curve; CI, confidence interval.

Based on the ELISA data of 52 diabetic patients and 55 normal controls in the validation group, ROC curves were established, and the auxiliary monitoring value of urinary exosome aerobic oxidative metabolism-related proteins was analyzed, as shown in **Figure 6**. The area under the curve (AUC) of urinary exosome GAPDH was 0.611 (95% CI, 0.503-0.718), and the AUC of urinary exosome PFKM was 0.714 (95% CI, 0.618-0.811). The AUC of urinary exosomal ACO2 was 0.650 (95% CI, 0.544-0.756), and AUC of urine exosomal MDH2 was 0.777 (95% CI, 0.685~0.868). When these 4 proteins were used in combination, the AUC reached 0.840 (95% CI, 0.764-0.915), which was more valuable than single use. In conclusion, urinary exosomal aerobic oxidative metabolism-related proteins may be potential biomarkers for monitoring diabetes and perform better when used in combination.

## Discussion

Diabetes mellitus is a metabolic defect disease with hyperglycemia as the main symptom, mainly due to decreased insulin secretion and insulin resistance. The study found that decreased insulin secretion may be mediated by abnormal glucose metabolism in beta cells, which is related to insulin biosynthesis and secretion (25). The role of glucose metabolism in the development of diabetes is still unclear. Glucose metabolic pathways include aerobic oxidation, anaerobic glycolysis, and glycogen formation, and abnormalities in each pathway may lead to the onset of diabetes. This study is the first to report the expression changes of aerobic oxidative metabolism proteins in the urinary exosomes of diabetic patients. This helps to explore the impact of aerobic oxidation processes on the pathogenesis of diabetes.

Through this study, we found and identified 17 proteins related to aerobic oxidative metabolism of glucose in diabetic urinary exosomes, and screened 5 proteins with differential expression, including PFKM, GAPDH, ACO2, MDH2, and IDH3G. Through ELISA, this study verified that the concentration expression of PFKM, GAPDH, ACO2, and MDH2 in the urine exosomes of diabetic patients was decreased, which was consistent with the protein changes observed by mass spectrometry. The aerobic oxidative metabolism-related proteins in urinary exosomes can be used as potential biomarkers for monitoring diabetes, and the monitoring value is even greater when these four proteins are used in combination. The application of aerobic oxidative metabolism-related proteins in urinary exosomes has unique advantages, which can be noninvasive, fast and simple. Combined monitoring with FBG will help further screening of diabetes.

Through ELISA experiments, we further explored and found that the concentrations of PFKM, GAPDH, and ACO2 in urinary exosomes had a strong positive correlation with the expression of MDH2. Aerobic oxidation is a continuous process, and abnormalities at each stage may lead to disease. Aerobic oxidative metabolism catalytic enzymes, as important catalytic enzymes of sugar metabolism, have many connections with each other. There was a significant positive correlation among PFKM, GAPDH, ACO2, and MDH2 in urinary exosomes, which further verifies the connection. The expression changes of PFKM, GAPDH, ACO2, and MDH2 in urinary exosomes may be closely related to the glucose metabolism activities of the body, but their roles are still unclear.

Urine acts as an ultrafiltrate of plasma and is the end product of metabolism in organs throughout the body (26, 27). Changes in urinary exosome expression should be consistent with systemic protein expression. We further explored the protein concentration and expression of PFKM, GAPDH, ACO2, and MDH2 in normal human urine exosomes of different ages. The expression of the four proteins showed a peak trend, and the expression level was the highest in the stage of 45-59 years old. Sander et al. performed a comprehensive *in vivo* quantitative proteomic analysis of islets from juvenile and 1-year-old mice and found that islets from adult mice exhibited enhanced levels and activities of glucose-metabolizing enzymes. By detecting the key enzymes in the pyruvate-citric acid cycle and the pyruvate-malate cycle, Pcx and Me1, the authors found that the enzyme activity had a significantly enhanced trend, and then further found that under the stimulation of glucose, the NADPH content increased, and the ratio of NADPH/NADP+ was higher in islets of adult mice compared to young mice (28). This is consistent with the results of this experiment. Before the age of 45, the expression and activity of carbohydrate aerobic oxidation-related proteins increased, the levels of metabolism-related proteins increased, and aerobic oxidative metabolism reached the highest level in the age group of 45 to 59 years old. Later, with increasing age, the insulin secretion of elderly individuals decreases, and the sensitivity of pancreatic beta cells to glucose is impaired, so the aerobic oxidative metabolism of glucose in elderly individuals is also significantly reduced, which may also be one of the reasons why the elderly are prone to diabetes (21).

Studies have found that gender differences may be an important factor in individual differences in proteomics. Gao’s team identified nine sex-related proteins by analyzing urine samples from healthy adults (29). Five of them were used to build models that could accurately distinguish between men and women. Unfortunately, we did not find significant gender differences in these four proteins in the validation cohort. In the future, gender will be a key factor in experimental design and data analysis in biomarkers. We will conduct more studies to investigate sex differences in aerobic oxidative metabolism-related proteins in urinary exosomes.

Given that the proteins PFKM, GAPDH, ACO2, and MDH2 have rate-limiting effects on aerobic oxidative metabolism, the decreased expression is consistent with the results of glucose metabolism disorders in diabetic patients. Mutations in muscle-type phosphofructokinase (PFKM) have been shown to cause glycogen storage disease VII (Tarui disease). It is an autosomal recessive metabolic disorder clinically characterized by exercise intolerance, muscle spasms, myopathy, and compensatory hemolysis (30, 31). The expression of PFKM decreased in senescent bone marrow mesenchymal cells (MSCs), and the overexpressed PFKM in turn upregulated the level of glucose metabolism in senescent MSCs and inhibited cell senescence. Notably, enhanced expression of 6-phosphofructokinase-2 isoenzyme 3 (PFKFB3) has been shown to be involved in improving insulin sensitivity and anti-inflammatory effects in obese mice (32). The product of GAPDH is an important enzyme in carbohydrate metabolism. The interaction between the glycolytic protein GAPDH and the inflammatory protein small ubiquitin-like modifier 4 has been shown to induce insulin resistance in diabetic and obese individuals (33). Malate dehydrogenase MDH2 is localized to mitochondria and utilizes the NAD/NADH cofactor system in the tricarboxylic acid cycle to catalyze the reversible oxidation of malate to oxaloacetate. A study identified MDH2 as a novel diabetes gene whose heterozygous mutation causes hyperglycemia in families with multigenerational diabetes (34, 35). In general, as enzymes directly involved in the aerobic oxidative metabolism of glucose, these four proteins with reduced expression may be related to the occurrence of diabetes, and how to play their roles needs to be further explored.

Prediabetes is a high-risk state of diabetes, and 5% - 10% of prediabetic patients will develop diabetes every year. It is particularly important to assess the risk state of prediabetes (36). In this experiment, we found that the expression of GAPDH, PFKM, and ACO2 in urinary exosomes was already reduced in prediabetes, although the difference was not significant (*p*>0.05), see **Supplementary Materials 4** for details. We speculate that these four protein changes in urinary exosomes have already appeared in prediabetes, but the prediabetes population in this study was small (n=14), resulting in the final difference being insignificant. This is the insufficiency of this study, and it is also the focus of our future research. Identifying protein changes in prediabetes could provide additional support for our biomarker research.

This experiment has some other shortcomings. First, the sample size is small and more samples need to be collected for analysis. The clinical value of urinary exosome aerobic oxidation-related proteins needs to be further evaluated and validated by expanding the sample size in a multicenter setting. At present, the protein concentration changes of the four proteins PFKM, GAPDH, ACO2, and MDH2 in urinary exosomes are only preliminary explorations. We have not determined which processes these proteins are related to, and more in-depth molecular pathway analysis is needed in the future.

In conclusion, in this study, we analyzed the expression trends of aerobic oxidative metabolism-related proteins in the urinary exosomes of diabetic patients and normal people of different ages. Metabolic disorders in patients with diabetes, decreased concentrations of aerobic oxidative metabolism-related proteins in urinary exosomes may lead to diabetes, which is helpful to explore the pathogenesis of diabetes from the perspective of aerobic oxidation. The differential expression of PFKM, GAPDH, ACO2, and MDH2 in the urinary exosomes of diabetic patients can serve as potential biomarkers for diabetes monitoring, providing a promising method for noninvasive diabetes diagnosis.

## Data availability statement

The data analyzed in this study is subject to the following licenses/restrictions: The datasets used and analyzed during the current study are available from the corresponding author on reasonable request. Requests to access these datasets should be directed to zhangman@bjsjth.cn.

## Ethics statement

This experiment was approved by the Ethics Committee of Beijing Shijitan Hospital. Written informed consent to participate in this study was provided by the participants’ legal guardian/next of kin.

## Author contributions

MZhan: Conceptualization, funding acquisition, project administration, resources, supervision. TCL: Formal analysis, investigation, validation, writing original draft. YW, MZhao, JJ, and TL: Investigation, validation. All authors read and approved the final manuscript.

## Funding

Validation and application development of new urine diagnostic and monitoring marker test in type 2 diabetes-related diseases (Z211100002921040).

## Acknowledgments

Thanks are due to all the volunteers for their generous donation of urine samples.

## Conflict of interest

The authors declare that the research was conducted in the absence of any commercial or financial relationships that could be construed as a potential conflict of interest.

## Publisher’s note

All claims expressed in this article are solely those of the authors and do not necessarily represent those of their affiliated organizations, or those of the publisher, the editors and the reviewers. Any product that may be evaluated in this article, or claim that may be made by its manufacturer, is not guaranteed or endorsed by the publisher.

## Supplementary material

SUPPLEMENTARY MATERIALS 1 The result of urinary exosome concentrations by NTA.

SUPPLEMENTARY MATERIALS 2 Clinical characteristics of healthy people of different ages.

SUPPLEMENTARY MATERIALS 3 Differential analysis of target proteins expression between males and females. **(A)** Expression analysis of four proteins in diabetic males (DM male, n=26) and diabetic females (DM female, n=26). The pictures are GAPDH, PFKM, ACO2, MDH2 in order. **(B)** Expression analysis of the four proteins in normal males (NC male, n=28) and normal females (NC female, n=27). The pictures are GAPDH, PFKM, ACO2, MDH2 in order.

SUPPLEMENTARY MATERIALS 4 Analysis of ELISA results in diabetic group (DM, n=52), normal control group (NC, n=41) and prediabetes (Prediabetic, n=14). The expressions of GAPDH **(A)**, PFKM **(B)**, ACO2 **(C)** and MDH2 **(D)** in urinary exosomes were measured by ELISA in three groups of people. The unit is ng/10μg, which represents the target protein content per 10μg of total protein. Symbols represent individual subjects, each measured once in an independent experiment. ***, p<0.001; **, p<0.01; *, p<0.05; ns, not significant.

## References

[1] Tao Z Shi A Zhao J. Epidemiological perspectives of diabetes. Cell Biochem biophysics (2015) 73(1):181–5. doi: 10.1007/s12013-015-0598-4 25711186

[2] Sun H Saeedi P Karuranga S Pinkepank M Ogurtsova K Duncan BB. IDF diabetes atlas: Global, regional and country-level diabetes prevalence estimates for 2021 and projections for 2045. Diabetes Res Clin Pract (2022) 183:109119. doi: 10.1016/j.diabres.2021.109119 34879977 PMC11057359

[3] Zethelius B Hales CN Lithell HO Berne C. Insulin resistance, impaired early insulin response, and insulin propeptides as predictors of the development of type 2 diabetes: a population-based, 7-year follow-up study in 70-year-old men. Diabetes Care (2004) 27(6):1433–8. doi: 10.2337/diacare.27.6.1433 15161800

[4] Basu R Breda E Oberg AL Powell CC Dalla Man C Basu A. Mechanisms of the age-associated deterioration in glucose tolerance: contribution of alterations in insulin secretion, action, and clearance. Diabetes (2003) 52(7):1738–48. doi: 10.2337/diabetes.52.7.1738 12829641

[5] Quadri Z Elsherbini A Bieberich E. Extracellular vesicles in pharmacology: Novel approaches in diagnostics and therapy. Pharmacol Res (2022) 175:105980. doi: 10.1016/j.phrs.2021.105980 34863822 PMC8760625

[6] Johnstone R. Maturation of reticulocytes: formation of exosomes as a mechanism for shedding membrane proteins. Biochem Cell Biol (1992) 70(3-4):179–90. doi: 10.1139/o92-028 1515120

[7] Colombo M Raposo G Théry C. Biogenesis, secretion, and intercellular interactions of exosomes and other extracellular vesicles. Annu Rev Cell Dev Biol (2014) 30:255–89. doi: 10.1146/annurev-cellbio-101512-122326 25288114

[8] Subra C Laulagnier K Perret B Record M. Exosome lipidomics unravels lipid sorting at the level of multivesicular bodies. Biochimie (2007) 89(2):205–12. doi: 10.1016/j.biochi.2006.10.014 17157973

[9] Buschow SI Liefhebber JM Wubbolts R Stoorvogel W. Exosomes contain ubiquitinated proteins. Blood Cells Molecules Dis (2005) 35(3):398–403. doi: 10.1016/j.bcmd.2005.08.005 16203162

[10] Kowal J Arras G Colombo M Jouve M Morath JP Primdal-Bengtson B. Proteomic comparison defines novel markers to characterize heterogeneous populations of extracellular vesicle subtypes. Proc Natl Acad Sci (2016) 113(8):E968–E77. doi: 10.1073/pnas.1521230113 PMC4776515 26858453

[11] Lu Y Liu D Feng Q Liu Z. Diabetic nephropathy: perspective on extracellular vesicles. Front Immunol (2020) 11:943. doi: 10.3389/fimmu.2020.00943 32582146 PMC7283536

[12] Menon R Dixon CL Sheller-Miller S Fortunato SJ Saade GR Palma C. Quantitative proteomics by SWATH-MS of maternal plasma exosomes determine pathways associated with term and preterm birth. Endocrinology (2019) 160(3):639–50. doi: 10.1210/en.2018-00820 PMC6388657 30668697

[13] Sheller-Miller S Trivedi J Yellon SM Menon R. Exosomes cause preterm birth in mice: evidence for paracrine signaling in pregnancy. Sci Rep (2019) 9(1):1–18. doi: 10.1038/s41598-018-37002-x 30679631 PMC6345869

[14] Safdar A Saleem A Tarnopolsky MA. The potential of endurance exercise-derived exosomes to treat metabolic diseases. Nat Rev Endocrinol (2016) 12(9):504–17. doi: 10.1038/nrendo.2016.76 27230949

[15] Carrière J Barnich N Nguyen HTT. Exosomes: from functions in host-pathogen interactions and immunity to diagnostic and therapeutic opportunities. Rev Physiology Biochem Pharmacol (2016) 172:39–75. doi: 10.1007/112_2016_7 27600934

[16] Kranendonk ME Visseren FL van Balkom BW Nolte-'t Hoen EN van Herwaarden JA de Jager W. Human adipocyte extracellular vesicles in reciprocal signaling between adipocytes and macrophages. Obesity (2014) 22(5):1296–308. doi: 10.1002/oby.20679 24339422

[17] Su T Xiao Y Xiao Y Guo Q Li C Huang Y. Bone marrow mesenchymal stem cells-derived exosomal MiR-29b-3p regulates aging-associated insulin resistance. ACS nano (2019) 13(2):2450–62. doi: 10.1021/acsnano.8b09375 30715852

[18] Guay C Menoud V Rome S Regazzi R. Horizontal transfer of exosomal microRNAs transduce apoptotic signals between pancreatic beta-cells. Cell communication Signaling (2015) 13(1):1–12. doi: 10.1186/s12964-015-0097-7 25880779 PMC4371845

[19] Bouche C Serdy S Kahn CR Goldfine AB. The cellular fate of glucose and its relevance in type 2 diabetes. Endocrine Rev (2004) 25(5):807–30. doi: 10.1210/er.2003-0026 15466941

[20] Kalra S Sharma SK. Diabetes in the elderly. Diabetes Ther (2018) 9(2):493–500. doi: 10.1007/s13300-018-0380-x 29460258 PMC6104259

[21] Chentli F Azzoug S Mahgoun S. Diabetes mellitus in elderly. Indian J Endocrinol Metab (2015) 19(6):744. doi: 10.4103/2230-8210.167553 26693423 PMC4673801

[22] Foers AD Chatfield S Dagley LF Scicluna BJ Webb AI Cheng L. Enrichment of extracellular vesicles from human synovial fluid using size exclusion chromatography. J extracellular vesicles (2018) 7(1):1490145. doi: 10.1080/20013078.2018.1490145 29963299 PMC6022248

[23] Mol EA Goumans M-J Doevendans PA Sluijter JP Vader P. Higher functionality of extracellular vesicles isolated using size-exclusion chromatography compared to ultracentrifugation. Nanomedicine: Nanotechnology Biol Med (2017) 13(6):2061–5. doi: 10.1016/j.nano.2017.03.011 28365418

[24] Takov K Yellon DM Davidson SM. Comparison of small extracellular vesicles isolated from plasma by ultracentrifugation or size-exclusion chromatography: yield, purity and functional potential. J extracellular vesicles (2019) 8(1):1560809. doi: 10.1080/20013078.2018.1560809 30651940 PMC6327926

[25] Leibiger B Moede T Schwarz T Brown GR Köhler M Leibiger IB. Short-term regulation of insulin gene transcription by glucose. Proc Natl Acad Sci (1998) 95(16):9307–12. doi: 10.1073/pnas.95.16.9307 PMC21334 9689076

[26] Thongboonkerd V Malasit P. Renal and urinary proteomics: current applications and challenges. Proteomics (2005) 5(4):1033–42. doi: 10.1002/pmic.200401012 15669002

[27] Zhou L-T Lv L-L Liu B-C. Urinary biomarkers of renal fibrosis. Renal Fibrosis: Mech Therapies (2019) 1165(1165), 607–23. doi: 10.1007/978-981-13-8871-2_30 31399987

[28] Wortham M Benthuysen JR Wallace M Savas JN Mulas F Divakaruni AS. Integrated *In Vivo* quantitative proteomics and nutrient tracing reveals age-related metabolic rewiring of pancreatic β cell function. Cell Rep (2018) 25(10):2904–18.e8. doi: 10.1016/j.celrep.2018.11.031 30517875 PMC6317899

[29] Shao C Zhao M Chen X Sun H Yang Y Xiao X. Comprehensive analysis of individual variation in the urinary proteome revealed significant gender differences. Mol Cell Proteomics (2019) 18(6):1110–22. doi: 10.1074/mcp.RA119.001343 PMC6553935 30894400

[30] Brüser A Kirchberger J Schöneberg T. Altered allosteric regulation of muscle 6-phosphofructokinase causes tarui disease. Biochem Biophys Res Commun (2012) 427(1):133–7. doi: 10.1016/j.bbrc.2012.09.024 22995305

[31] Toscano A Musumeci O. Tarui disease and distal glycogenoses: clinical and genetic update. Acta Myologica (2007) 26(2):105. 18421897 PMC2949577

[32] Huo Y Guo X Li H Xu H Halim V Zhang W. Targeted overexpression of inducible 6-phosphofructo-2-kinase in adipose tissue increases fat deposition but protects against diet-induced insulin resistance and inflammatory responses. J Biol Chem (2012) 287(25):21492–500. doi: 10.1074/jbc.M112.370379 PMC3375570 22556414

[33] Sengupta U Ukil S Dimitrova N Agrawal S. Expression-based network biology identifies alteration in key regulatory pathways of type 2 diabetes and associated risk/complications. PloS One (2009) 4(12):e8100. doi: 10.1371/journal.pone.0008100 19997558 PMC2785475

[34] Jungtrakoon P Pezzilli S Pezzilli A Pannone L Flex E Biagini T. Malate dehydrogenase 2 (MDH2) as a new diabetogene causing hyperglycemia in families with multigenerational diabetes. Diabetes (2018) 67(Supplement_1). doi: 10.2337/db18-262-OR

[35] Jungtrakoon Thamtarana P Marucci A Pannone L Bonnefond A Pezzilli S Biagini T. Gain of function of malate dehydrogenase 2 and familial hyperglycemia. J Clin Endocrinol Metab (2022) 107(3):668–84. doi: 10.1210/clinem/dgab790 PMC8852227 34718610

[36] Tabák AG Herder C Rathmann W Brunner EJ Kivimäki M. Prediabetes: a high-risk state for diabetes development. Lancet (2012) 379(9833):2279–90. doi: 10.1016/S0140-6736(12)60283-9 PMC3891203 22683128

