# Supplementary figures and images for: Differential expression of aerobic oxidative metabolism-related proteins in diabetic urinary exosomes
Source: Front Endocrinol (Lausanne). 2022 Sep 14;13:992827. doi: 10.3389/fendo.2022.992827 (PMC9515495; doi:10.3389/fendo.2022.992827)

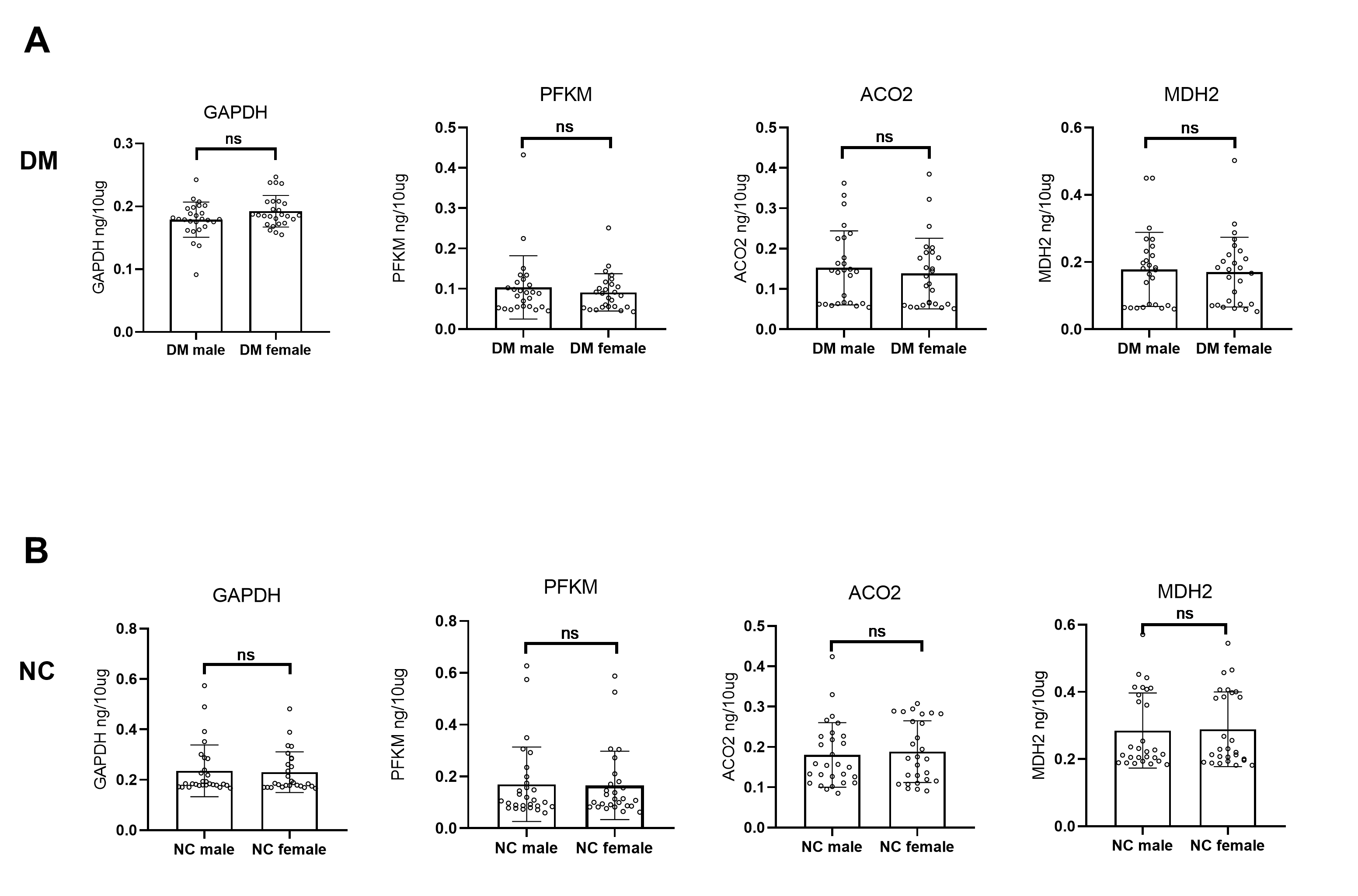

Supplement: SUPPLEMENTARY MATERIALS 1 — The result of urinary exosome concentrations by NTA. [file Image_1.tif]

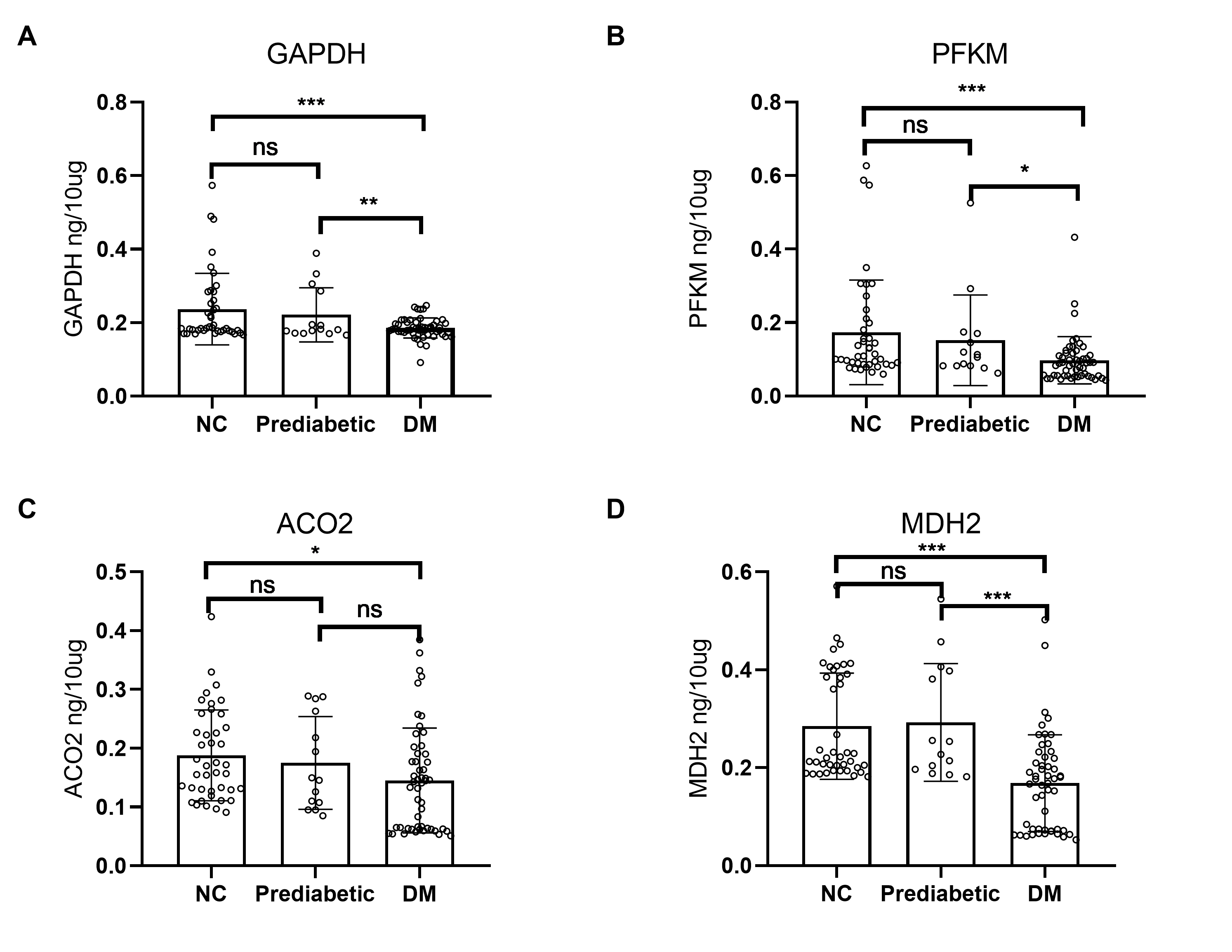

Supplement: SUPPLEMENTARY MATERIALS 2 — Clinical characteristics of healthy people of different ages. [file Image_2.tif]
