# Supplementary material for: Differential expression of aerobic oxidative metabolism-related proteins in diabetic urinary exosomes
Source: Front Endocrinol (Lausanne). 2022 Sep 14;13:992827. doi: 10.3389/fendo.2022.992827 (PMC9515495; doi:10.3389/fendo.2022.992827)
Supplement: SUPPLEMENTARY MATERIALS 3 — Differential analysis of target proteins expression between males and females. (A) Expression analysis of four proteins in diabetic males (DM male, n=26) and diabetic females (DM female, n=26). The pictures are GAPDH, PFKM, ACO2, MDH2 in order. (B) Expression analysis of the four proteins in normal males (NC male, n=28) and normal females (NC female, n=27). The pictures are GAPDH, PFKM, ACO2, MDH2 in order. [file Table_1.docx]

**Table S1** **Expression analysis of aerobic oxidative metabolic proteases in urinary exosomes**

| **Uinorot-ID** | **Protein Name** | **Gene Name** | **FC** | **P-value** | **Form of expression** |
| --- | --- | --- | --- | --- | --- |
| P08237 | ATP-dependent 6-phosphofructokinase, muscle type | PFKM | 0.42 | 1.70E-05 | down |
| P04406 | Glyceraldehyde-3-phosphate dehydrogenase | GAPDH | 0.59 | 9.00E-06 | down |
| Q99798 | Aconitate hydratase, mitochondrial | ACO2 | 0.35 | 3.74E-03 | down |
| P40926 | Malate dehydrogenase, mitochondrial | MDH2 | 0.27 | 8.98E-03 | down |
| P51553 | Isocitrate dehydrogenase [NAD] subunit gamma, mitochondrial | IDH3G | 4.08 | 4.35E-02 | up |
| P52790 | Hexokinase-3 | HK3 | 0.84 | 0.56038 | n.s. |
| P17858 | ATP-dependent 6-phosphofructokinase, liver type | PFKL | 0.68 | 0.000099 | n.s. |
| P00558 | Phosphoglycerate kinase 1 | PGK1 | 0.75 | 0.002712 | n.s. |
| P14618 | Pyruvate kinase PKM | PKM | 0.88 | 0.406739 | n.s. |
| P24752 | Acetyl-CoA acetyltransferase, mitochondrial | ACAT1 | 0.75 | 0.142086 | n.s. |
| Q9BWD1 | Acetyl-CoA acetyltransferase, cytosolic | ACAT2 | 0.75 | 0.467052 | n.s. |
| P53396 | ATP-citrate synthase | ACLY | 0.55 | 0.092287 | n.s. |
| P21399 | Cytoplasmic aconitate hydratase | ACO1 | 0.73 | 0.027635 | n.s. |
| O75874 | Isocitrate dehydrogenase [NADP] cytoplasmic | IDH1 | 0.70 | 0.18293 | n.s. |
| P48735 | Isocitrate dehydrogenase [NADP], mitochondrial | IDH2 | 0.85 | 0.460747 | n.s. |
| P36957 | Dihydrolipoyllysine-residue succinyltransferase component of 2-oxoglutarate dehydrogenase complex, mitochondrial | DLST | 0.75 | 0.508987 | n.s. |
| P40925 | Malate dehydrogenase, cytoplasmic | MDH1 | 0.76 | 0.159265 | n.s. |
