# Supplementary material for: Differential expression of aerobic oxidative metabolism-related proteins in diabetic urinary exosomes
Source: Front Endocrinol (Lausanne). 2022 Sep 14;13:992827. doi: 10.3389/fendo.2022.992827 (PMC9515495; doi:10.3389/fendo.2022.992827)
Supplement: SUPPLEMENTARY MATERIALS 4 — Analysis of ELISA results in diabetic group (DM, n=52), normal control group (NC, n=41) and prediabetes (Prediabetic, n=14). The expressions of GAPDH (A), PFKM (B), ACO2 (C) and MDH2 (D) in urinary exosomes were measured by ELISA in three groups of people. The unit is ng/10μg, which represents the target protein content per 10μg of total protein. Symbols represent individual subjects, each measured once in an independent experiment. ***, p<0.001; **, p<0.01; *, p<0.05; ns, not significant. [file Table_2.docx]

**Supplementary materials 2.** **Clinical characteristics of healthy people of different ages**

| Characteristics | 0~6  (n=20) | 7~14  (n=20) | 15~30  (n=20) | 31~44  (n=20) | 45~59  (n=20) | 60~79  (n=20) | ≥80  (n=20) |
| --- | --- | --- | --- | --- | --- | --- | --- |
| Age, years | 3.55±0.30 | 11.05±0.46 | 24.00±0.94 | 38.85±0.84 | 52.00±0.99 | 66.00±1.28 | 87.75±1.02 |
| Gender,  men/female | 10/10 | 10/10 | 10/10 | 10/10 | 10/10 | 10/10 | 10/10 |
| Weight, kg | NA | 45.58±1.17 | 57.13±1.59 | 63.60±1.82 | 64.25±1.61 | 63.31±1.47 | 60.23±1.67 |
| BMI | NA | 17.97±0.43 | 20.11±0.40 | 22.47±0.39 | 23.22±0.44 | 22.84±0.20 | 21.67±0.45 |
| WBC×10^9^/L | 5.59±0.25 | 5.38±0.26 | 5.72±0.26 | 5.99±0.25 | 5.47±0.39 | 5.60±0.22 | 6.39±0.53 |
| RBC×10^12^/L | 4.26±0.12 | 4.39±0.11 | 4.81±0.11 | 4.84±0.07 | 4.78±0.08 | 4.35±0.13 | 3.79±0.14 |
| Hb, g/L | 121.60±3.07 | 125.8±2.56 | 141.80±3.47 | 145.00±2.76 | 141.40±4.09 | 125.60±3.90 | 113.10±4.60 |
| Cr, μmol/L | 31.25±1.56 | 41.05±1.92 | 72.80±2.90 | 67.95±2.87 | 69.10±2.71 | 61.70±3.41 | 86.10±3.60 |
| eGFR,mL/min/1.73m2 | 160.00±3.82 | 149.70±2.01 | 111.70±2.76 | 110.20±1.99 | 99.55±1.57 | 87.85±3.61 | 58.85±4.58 |
| FBG, mmol/L | 4.79±0.08 | 5.04±0.06 | 5.00±0.10 | 5.19±0.08 | 5.28±0.08 | 5.41±0.10 | 5.53±0.19 |
| HbA1c, % | 4.94±0.05 | 5.11±0.05 | 5.32±0.06 | 5.36±0.03 | 5.48±0.04 | 5.38±0.05 | 5.45±0.07 |
| AST, U/L | 27.65±1.36 | 23.80±1.33 | 17.05±0.60 | 17.35±0.56 | 19.60±0.84 | 24.45±1.75 | 21.35±2.27 |
| ALT, U/L | 18.10±1.37 | 23.80±1.59 | 16.35±1.10 | 16.85±1.31 | 22.00±2.12 | 22.10±1.84 | 17.70±2.44 |
| Urine protein; (negative, %) | 20（100） | 20（100） | 20（100） | 20（100） | 20（100） | 20（100） | 20（100） |
| Comorbidities | No | No | No | No | No | No | No |

**Note:** BMI: Body Mass Index; WBC: White blood cell; RBC: Red blood cell; Hb: Hemoglobin; Cr: Creatinine; eGFR: Estimated glomerular filtration rate; FBG: Fasting Blood Glucose; AST: Aspartate aminotransferase; ALT: Alanine aminotransferase.
