# Supplementary material for: Differential expression of aerobic oxidative metabolism-related proteins in diabetic urinary exosomes
Source: Front Endocrinol (Lausanne). 2022 Sep 14;13:992827. doi: 10.3389/fendo.2022.992827 (PMC9515495; doi:10.3389/fendo.2022.992827)
Supplement: Supplementary file 5 [file DataSheet_1.pdf]

Electrophoresis & Brownian Motion
Video Analysis
Laser Scattering Microscopy

www.particle-metrix.de

Operator (Report): Administrator
Video Operator: Administrator

Sample Parameters
Sample Name: 206
Comment: 1:10, Sample Remarks0:
Sample Remarks1:
Sample Remarks2:
Electrolyte: h2o
Temperature: 26.82 °C sensed
pH 7.4 entered
Conductivity: 0.00 µS/cm entered

Result (sizes in nm)

	Number	Concentration	Volume
Median (X50)	118.5	118.5	244.0
Span	72.9	72.9	140.5

Concentration: 2.9E+8 Particles / mL
Dilution Factor: 100
Original Concentration: 2.9E+10 Particles / mL

Quality
Average Counted Particles per Frame: 638
Number of Traced Particles: 1783

Measurement Parameters
Cell S/N: NTA

Measurement Mode: Size Distribution 4 Cycles
11 Positions, 2 Removed for Analysis

Analysis Parameters
Max Area: 1000, Min Area: 10, Min Brightness: 30

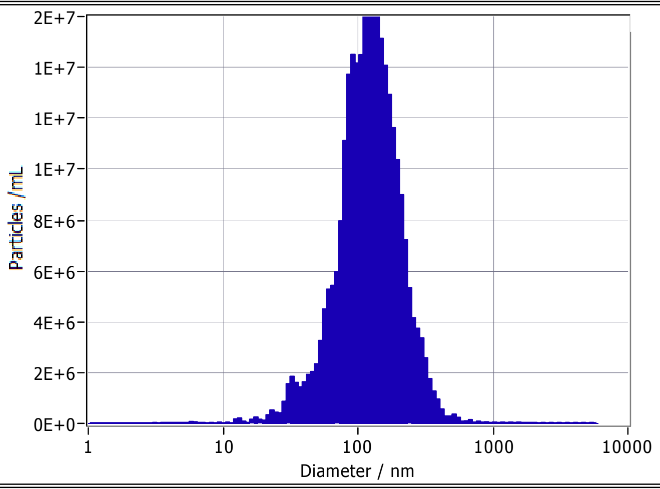

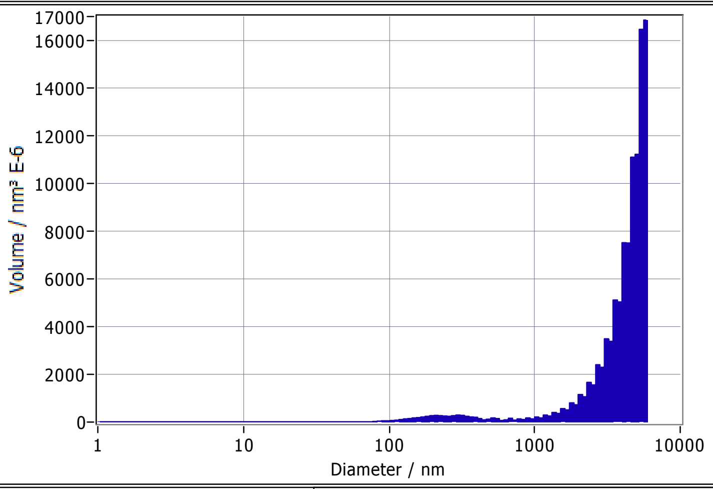

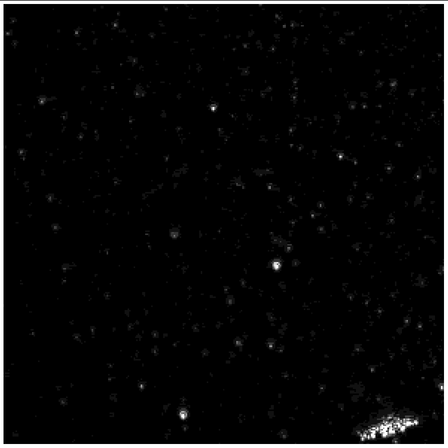

Peak Analysis (Concentration)

Diameter / nm	Particles/mL	FWHM / nm	Percentage
126.3	1.8E+7	135.8	99.3
13.2	2.1E+5	1.3	0.3
1630.3	3.8E+4	136.7	0.1
3.1	3.2E+4	2.2	0.1
2923.9	3.0E+4	239.3	0.1

X Values

	Number	Concentration	Volume
X10	59.0	59.0	128.5
X50	118.5	118.5	244.0
X90	217.3	217.3	494.7
Span	1.3	1.3	1.5
Mean	136.1	136.1	284.9
StdDev	72.9	72.9	140.5

Comment

(Signature)

Analyzed Video: E:\SXD-NTA\20201224\tmz-u87-zjw-exo-2\20220321_0001_dpmsc1_size.avi

ZetaVIEW S/N 17-340, Software ZetaView 8.04.02, Camera 0.714 µm/px

Experiment: 2022-03-21 19:08, Report: 2022-03-21 19:38
